# Navalis, Jun. on Burns and Scalds

**Published:** 1801-03

**Authors:** 

**Affiliations:** Spithead


### *To the Editors of the Medical and Phyſical Journal.*

Gentlemen,

WHEN the application of any new remedy is recommended to the Public, it may be conſidered as a duty incumbent on every member of the Faculty, to make known any facts he is acquainted with, whether for or againſt it. Having ſeen the application of the ſpt. terebinth. in caſes of Burns and Scalds recommended by ſome of your ingenious correſpondents in different Numbers of this Work, I have, for the above reaſon, taken this method of ſubmitting to your opinion, the effects which I have obſerved enſue from its uſe in a variety of caſes of the above kind. The practice is, I believe, by ſome, conſidered new; but it is certainly by no means entirely ſo, for it has been known and uſed many years in the neighbourhood of ſome of our manufacturing towns, where the nature of the trade is ſuch, as to render the workmen particularly liable to injuries by fire. At leaſt, I am authoriſed to ſay, this is the caſe with reſpect to Birmingham, Wolverhampton, and Dudley, from reſiding nearly ſeven years in the vicinity of thoſe towns, where the common people are ſo well acquainted with the good conſequences following its early application, that it is commonly, and almoſt conſtantly, kept in their work-ſhops. During the above period, which was from 1791 to 1798, I had opportunities of ſeeing above 120 caſes of injuries by the exceſſive application of heat, different in degrees of ſeverity; in above 100 of which, the ſpt. terebinth. was always applied, whenever ſurgical aſſiſtance was called in during the period that it could be deemed uſeful. The hint of its utility was firſt derived from the lower claſs of mechanics, who, as I have before obſerved, had been long in the habit of uſing it. I am extremely ſorry it is out of my power to lay any of theſe caſes before the public; from having left that part of the country in the year laſt mentioned, and having been ſince for the moſt part employed out of the kingdom, I am entirely unfurniſhed with the names and exact dates;—this will, I hope, be conſidered as a ſufficient excuſe, as no practical concluſions can be deduced from the recital of caſes, unleſs their authenticity and and accuracy can be depended upon in every particular. The following, however, are the general effects which I have commonly obferved from its ufe:

That, if applied immediately, or foon after the injury is fuftained, the excruciating pain is always alleviated, and often removed; the period in which this happens, is generally in proportion to its early or late application.

That its beneficial effects are moft evident in thofe cafes where the injury penetrates no deeper than the cutis; and it is generally obferved, the moft violent degrees of pain and irritation happen in fuch inftances. Confequently, it will be found commonly lefs ufeful in burns than in fcalds.

That it will, notwithftanding, be found of the greateft fervice in burns, even of the fevereft kind, in which the chief pain is always found at the junction of the injured and found parts; where, by allaying the irritation, it often prevents violent degrees of inflammation in the adjacent parts, and thus forwards the feparation of the efchar.

That its utility in fuperficial injuries appears to be by preventing effufion and confequent ulceration; and in fuch cafes, its beneficial application may be limited to within twenty-four hours after the injury is received; but in feverer cafes it may be ufefully applied, until fuch time as floughing commences, and fuppuration is eftablifhed.

The mode of its application is the next confideration.—It may be applied alone by means of a feather, or large camel-hair brufh; or cloths thoroughly wetted with it, may be applied over the affected parts: But though, in this way, it acts quickly in removing pain after the irritation immediately fubfequent to its application is over, I am inclined to think its falutary effects are not fo permanent as when mixed with an equal quantity of fine olive oil. Fine rags dipt in this liniment, and applied doubly, form a foft and eafy dreffing, which may, for the moft part, be removed without pain, and appear to retain their influence feveral hours; whereas, when ufed by itfelf, it foon evaporates, and the rags become ftiff, dry, and a caufe of irritation; or when mixed with linfeed oil, as is the practice with fome, after lying on a fhort time, they adhere fo faft as in their removal frequently to tear away the fkin, and thus effectually fruftrate the chief curative intention. The repetition may be every fix hours, or as often as the parts affected become very painful. That opiates are ufefully fubjoined, I need not mention.

In every topical affection it will be allowed, the prevention or removal of pain is a principal ftep towards cure; this is of particular moment in extenfive burns or fcalds fituate on the regions regions of the ftomach or abdomen, where exceffive irritation is likely to extend the inflammation to fome of the neighbouring vifcera, and thus deftroy life ; and in far the greateft number of accidents of the above nature, which happened to children from upfetting tea-pots, or their cloaths catching fire, thefe parts and the throat have fuffered moft ; at leaft, this has happened in moft of thofe I have happened to be engaged in ; but it was, probably, accidental, and may not in general be the cafe.

In the year 1797, I had an opportunity of feeing three cafes of fcalds treated by the application of cold, though not perfonally concerned in their treatment ; they all happened from boiling wort, and the fubjects were healthy unmarried women, all under 25 years of age. Two happened at the fame time, and were treated by the immediate application of cold cataplafins of fcraped potatoes, renewed as often as they became temperate ; one was above the right ancle, and the other on the left inftep : Though the injuries were by no means fevere, and the applications immediate, extenfive ulceration fucceeded in both cafes. The third was on the metatarfus, extending from the toes to the inftep. Immediately on being fcalded, fhe plunged her foot into a pail of cold water, where fhe kept it nearly half an hour ; the fimple liniment (ex ol. oliv. et aq. calcis) was afterwards applied ; but notwithftanding the attentive care of an eminent furgeon, extenfive floughing and ulceration enfued ; nor was fhe perfectly cured till near three months afterwards, during which time the bark, tonics, and opiates were found neceffary. I cannot pofitively affert that the application of the fpt. terebinth. would have prevented the difagreeable confequences in thefe three cafes ; but were I to draw conclufions from fimilar cafes, I fhould be induced to think it would.

I hope it will not be confidered, that the above fketch is by any means meant as a decifion in favour of the new practice ; I have merely narrated the effects I have obferved, with the opinion deduced from fuch obfervations ; and as I truft, by means of your valuable Journal, the affair will fhortly be decided by fome of your eminent correfpondents, whofe extenfive practice affords them numerous opportunities of making experiments, and from whofe abilities and integrity we have every reafon to hope for the moft juft and candid conclufions ; when this happens, if I find that I have been too hafty in forming my opinion on the fpt. terebinth. it will give me pleafure to adopt that which may be more conducive to the promotion of the healing art and the good of mankind. I am, &c.

*Spithead, Jan.* 25, 1801, NAVALIS, jun.

